# Relations of physical signs to genotype, lipid and inflammatory markers, coronary stenosis or calcification, and outcomes in patients with heterozygous familial hypercholesterolemia

**DOI:** 10.1186/s12967-021-03166-w

**Published:** 2021-12-07

**Authors:** Ming-Ming Liu, Jia Peng, Yuan-Lin Guo, Cheng-Gang Zhu, Na-Qiong Wu, Rui-Xia Xu, Qian Dong, Jian-Jun Li

**Affiliations:** Cardiovascular Metabolic Center, State Key Laboratory of Cardiovascular Diseases, Fu Wai Hospital, National Clinical Research Center for Cardiovascular Diseases, Chinese Academy of Medical Sciences and Peking Union Medical College, No. 167 BeiLiShi Road, XiCheng District, Beijing, China

**Keywords:** Tendon xanthomas, Corneal arcus, *LDLR* mutation, Heterozygous familial hypercholesterolemia

## Abstract

**Background:**

Although the presence of physical signs [tendon xanthomas and/or corneal arcus (TX/CA)], are associated with the risk of coronary artery disease in patients with heterozygous familial hypercholesterolemia (HeFH), their relationship with genotypes and clinical characteristics has not been fully determined. This study aimed to examine the association of TX/CA with genetic mutation, lipid- and inflammation-related markers, the severity of coronary stenosis or calcification, and cardiovascular events (CVEs) in patients with HeFH.

**Methods:**

*LDLR*, *APOB*, and *PCSK9* genes were screened in 523 HeFH patients, and patients with TX/CA (n = 50) were 1:4 propensity score-matched to patients without TX/CA (n = 200) to adjust for age and sex. Laboratory markers (proprotein convertase subtilisin/kexin type 9 [PCSK9], lipoprotein(a) and high-sensitivity C-reactive protein [hsCRP]), computed tomography angiography, coronary angiography, and follow-up for CVEs were performed.

**Results:**

Patients with physical signs had significantly higher low-density lipoprotein cholesterol levels; higher PCSK9 or hsCRP concentrations; more *LDLR* positive mutations; and higher prevalence of high tertiles of Gensini, SYNTAX and Jeopardy scores as well as coronary artery calcium scores than did those without. Over an average follow-up of 3.7 years, the incidence of CVEs was significantly higher in patients with TX/CA (log-rank *p* < 0.001). Patients with physical signs and mutation positivity had threefold higher risks of CVEs (adjusted hazard ratio 3.34, 95% confidence interval 1.04–10.72, *p* = 0.024).

**Conclusions:**

Physical signs were associated with genotypes and phenotypes, and worse outcomes in patients with HeFH, suggesting that these signs may help in risk stratification in these patients.

**Supplementary Information:**

The online version contains supplementary material available at 10.1186/s12967-021-03166-w.

## Introduction

Familial hypercholesterolemia (FH) is a genetic lipoprotein disorder characterized by an elevated plasma low-density lipoprotein cholesterol (LDL-C) level, deposition of cholesterol in extravascular tissues such as tendon xanthomas (TX) and corneal arcus (CA), and an increased risk of premature coronary artery disease (CAD) [1]. FH is mainly caused by pathological mutations, such as LDL receptor (*LDLR*), apolipoprotein B (*APOB*), and proprotein convertase subtilisin/kexin type 9 (*PCSK9*). The presence of physical signs (TX and/or CA) is the major criterion for the clinical diagnosis of heterozygous FH (HeFH). It has been postulated that TX/CA and atherosclerosis have similar pathogenic processes, featuring the accumulation of lipids in perivascular or avascular tissues such as the tendon sheath and corneal limbus. Interestingly, the physical signs vary based on the severity of hypercholesterolemia and CAD, even in subjects with the same LDL receptor gene defect [2].

Lipid- and inflammation-related markers, such as lipoprotein(a) [Lp(a)], PCSK9, and high-sensitivity C-reactive protein (hsCRP), play key roles in modulating atherosclerotic cardiovascular diseases, as indicated by genetic studies and clinical outcomes [3, 4]. PCSK9 binds to the LDL receptor, promoting degradation and thus elevating plasma LDL-C levels. Previous studies found that the plasma PCSK9 concentration was directly associated with the Lp(a) concentration, and PCSK9 inhibitors could reduce the Lp(a) concentration by approximately 25‒30% [5]. In addition, elevated Lp(a) levels are associated with an increased the risk of atherosclerotic cardiovascular diseases [6]. Inflammation has been recognized as a major mechanism of atherosclerotic lesion and CRP deposit formation in the arterial wall during atherosclerosis [7]. Several previous studies, including ours, have indicated that plasma PCSK9 levels are associated with inflammation-related markers [8]. This finding indicates that markers, such as Lp(a), PCSK9, and hsCRP, can also be potential predictors of cardiovascular risk, especially in high-risk individuals with HeFH.

TX and CA are both extravascular concomitants of atherosclerosis and are broadly associated with artery severity. The possible underlying mechanism could be that endothelial permeability allows macromolecular plasma proteins, such as LDL, to permeate the endothelium and interact with components of the arterial wall or tissues [9]. Several studies have reported the impact of physical signs (i.e. TX/CA) on CAD risk [10, 11]; in particular, Oosterveer et al. demonstrated that xanthomas was associated with a threefold higher risk of CVD in patients with FH [12]. These findings suggest that TX/CA has an impact on CAD risk; however, no studies have systematically evaluated the association between the presence of TX/CA and clinical characteristics/outcomes in patients with HeFH. We therefore performed a propensity score (PS)-matched cohort study to reveal the relationship between physical signs and genetic background, lipid- and inflammation-related markers, CAD severity, coronary calcification, and cardiovascular events (CVEs) in HeFH patients.

## Methods

### Study population

From January 2011 to September 2019, we analyzed data from 523 patients diagnosed with clinical FH, selected using the Dutch Lipid Clinic Network (DLCN) criteria from among 14,236 patients with suspected CAD who underwent elective coronary angiography at Fuwai Hospital. All the FH patients were screened for pathological mutations in *LDLR*, *APOB* and *PCSK9* genes, and they had a molecular and/or clinical DLCN score ≥ 6 (definite ≥ 8 and probable 6–8). Patients who had decompensated heart failure, severe hepatic or renal insufficiency, thyroid dysfunction, systemic inflammatory disease, malignant disease, homozygous FH, incomplete TX/CA recordings and were < 18 years of age or lost to follow-up were excluded. Thus, 466 patients with HeFH were included in the PS matching analysis. A flowchart illustrating study inclusion and exclusion is shown in Additional file 1: Fig. S1.

The study complied with the Declaration of Helsinki and was approved by the hospital’s ethical review board (Fu Wai Hospital & a Center for Cardiovascular Diseases, Beijing, China, Approval number: 2013-442). Informed written consents were obtained from all patients enrolled in this study.

### PS matching

**Fig. 1:**
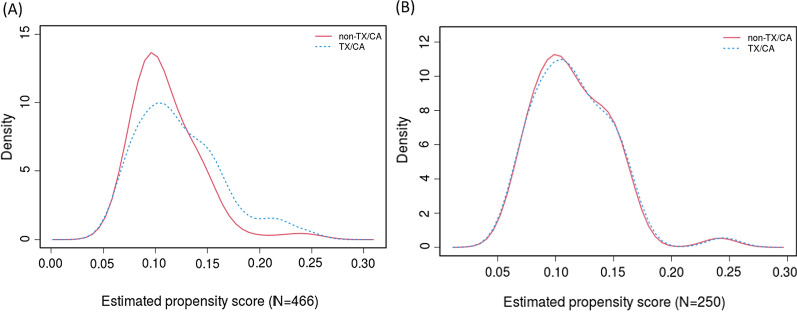
Distribution of the propensity score between TX/CA and non-TA/CA groups before (**A**) and after (**B**) propensity score matching. TX: tendon xanthomas; CA: corneal arcus

PS matching was performed using multivariable logistic regression models to reduce the impact of confounding factors due to age and sex differences in patients. Case-controlled PS matching without replacement was performed at a 1:4 ratio for TX/CA patients versus non-TX/CA patients to adjust for age and sex, with no significant difference and within a 10% standard deviation (SD) [13]. Optimal matching was used, and the calliper width was < 0.2 of the SD of the PSs. After PS matching for analysis, 50 patients with TX/CA and 200 patients without TX/CA were included in the post-propensity-matched patient cohorts. The study cohort was also divided into four subgroups based on physical signs and genetic mutations (Additional file 1: Fig. S1). The distribution and covariate balance of the PSs before and after matching are provided in Additional file 1: Table S1 and Fig. 1.

### Genetic and bioinformatic analysis

Whole blood samples were collected from patients with a probable or definite FH diagnosis (DLCN score ≥ 6) to perform a genetic test. Molecular analyses of mutations including 3 FH genes (*LDLR*, *APOB* and *PCSK9*) were performed using target exome sequencing which covered all the coding exons. The enriched DNA samples were sequenced using the Hiseq2000 Sequencing System (Illumina) [6]. The identified single nucleotide polymorphisms and the insertion or deletions were annotated using the Exome-assistant program. The functional effect of non-synonymous single nucleotide variant was predicted using Sorting Intolerant From Tolerant, Polymorphism Phenotyping and Mutation Taster. The deleteriousness of insertion/deletion variants were assessed using Combined Annotation Dependent Depletion, Dann and Eigen. A positive genetic diagnosis of FH was reported by the presence of at least one pathogenic or likely pathogenic variant of one allele of the candidate gene, or variants of both alleles for recessive forms.

### Laboratory analysis

Blood samples for assays were obtained from the cubital vein after overnight fasting. The concentrations of plasma total cholesterol (TC), triglyceride (TG), LDL-C and high-density lipoprotein cholesterol (HDL-C) were measured using an automatic biochemistry analyzer (Hitachi 7150, Tokyo, Japan). Lp(a) levels were assayed using an immunoturbidimetry method according to the manufacturer's instructions, as previously described [14]. HsCRP levels were determined using immunoturbidimetry (Beckmann Assay 360; Beckman Coulter, Brea, CA, USA). PCSK9 levels were determined using a high-sensitivity, quantitative sandwich enzyme immunoassay (Quantikine ELISA, R&D Systems Europe Ltd). Other related biochemical and hematological parameters were measured using standard tests.

### Definition of physical signs

TX was defined by the thickening of Achilles tendons or tendons on the backs of the hands, elbows, and knees, as determined by X-ray assessment or physical examination. Achilles tendon xanthomas was deemed as over a maximum of 9 mm thickness. CA was defined by a grey-white or yellow opacity located near the periphery of the cornea but separated from the limbus by a clear zone and a corneal arc of more than 180° [10]. Physical signs (TX/CA) were measured by two experienced physicians.

### Definition of clinical variables

Data for baseline characteristics of patients including demographic information, family and medical history, medication use, lifestyle factors, and revascularisation procedure-related factors were collected by experienced physicians and nurses. Family history of CAD was defined as myocardial ischemia or myocardial infarction (MI) documented in at least one first-degree relative. Family history of premature CAD was defined as first-degree relative with known premature CAD (at < 55 years for men and < 60 years for women) CAD. The body mass index (BMI) was calculated as weight in kilograms divided by height in meters squared. Smoking was defined as smoking at present (current smoker) or previously.

Hypertension was defined as a systolic blood pressure > 140 mmHg or a diastolic blood pressure > 90 mmHg on at least three occasions, currently taking antihypertensive drugs, or a self-reported history of hypertension based on hypertension guidelines [15]. Type 2 diabetes mellitus (T2DM) was defined as an fasting plasma glucose concentration ≥ 7.0 mmol/L, a 2 h plasma glucose concentration ≥ 11.1 mmol/L in an oral glucose tolerance test, a serum HbA1c not less than 6.5%, current use of hypoglycemic drugs or insulin, or a self-reported history of T2DM according to the American Diabetes Association definitions [16]. CAD was defined as 50% stenosis or greater in at least one major epicardial artery segment assessed by at least two experienced interventional cardiologists after coronary angiography. Information on baseline medications was collected via interviews or medical histories.

Uncontrolled LDL cholesterol levels were adjusted by the type and potency of lipid lowering drugs as previously described [17]. The cholesterol year score is a useful tool for examination of lifelong vascular exposure to elevated levels of lipoprotein cholesterol. The LDL-C year score was calculated as follows: LDL-C year score (mg-year/dL) = LDL-C max (mg/dL) at diagnosis * age at diagnosis + LDL-C (mg/dL) at inclusion * [age at inclusion – age at diagnosis]. The total cholesterol year score was calculated as: total cholesterol year score (mg-year/dL) = TC max (mg/dL) at diagnosis * age at diagnosis + TC (mg/dL) at inclusion * [age at inclusion – age at diagnosis] [18, 19].

### Evaluation of CAD severity and coronary calcification

Angiographic data were collected from catheter laboratory records during hospitalization. CAD severity was assessed using scoring systems, including Gensini, SYNTAX, and Jeopardy scores. The procedure was performed by at least two experienced interventional physicians. The Gensini score was used to define CAD severity based on the grade of stenosis, starting with 25% obstruction to total occlusion of the artery and including the anatomical location in the coronary circulation according to previous publications [20, 21]. The SYNTAX score was calculated using an online calculator (http://syntaxscore.org/). The Jeopardy score was determined using six segments, including the left anterior descending artery, the major anterolateral (diagonal) branch, the first major septal perforator, the left circumflex artery, the major circumflex marginal branch, and the posterior descending artery, each segment with a luminal diameter reduction > 75% was assigned a score of 2 points [22]. Tertiles of the above three scores were also established.

A total of 77 subjects who underwent coronary computed tomographic angiography (CCTA) were also evaluated to determine the coronary artery calcium (CAC) score. CCTA investigations were performed using a 64-slice scanner (Lightspeed VCT, GE Healthcare, Milwaukee, WI, USA). CAC was measured using the Agatston method and expressed as the coronary calcium score [23].

### Follow-up and outcomes

Patients were followed up semi-annually by direct or telephonic interview. Trained nurses or physicians who were blinded to the clinical data conducted the interview during the follow-up period. CVEs were defined as a composite of non-fatal MI, nonfatal ischemic stroke, coronary revascularization, and cardiac death. MI was defined as symptomatic chest pain, appearance of new Q waves on an electrocardiogram, and an elevated cardiac enzyme level. Incident ischemic stroke, excluding lacunar infarction, was defined as a new-onset stroke diagnosed using computed tomography or magnetic resonance imaging during the follow-up period. Coronary revascularization was defined as percutaneous coronary intervention or coronary artery bypass grafting, with or without stent replacement. Cardiac mortality was diagnosed using additional information from hospital records, death certificates, and family contacts. All CVEs were independently adjudicated by two investigators blinded to patient characteristic data.

### Statistical analysis

Values are expressed as mean ± SD or median (Q1–Q3 quartiles) for continuous variables and as numbers (percentages) for categorical variables. The Kolmogorov–Smirnov test was used to test the distribution pattern of the data. The χ2 test and *t*-test were used to compare characteristics between patients with and without TX/CA. Logistic regression models were used to examine the association between TX/CA and genetic mutations by calculating odds ratios (ORs) and 95% confidence intervals (CIs) for. A Kaplan–Meier curve was performed to determine the overall event-free survival time according to physical signs and mutation subgroups. Cox proportional hazard regression models were employed to evaluate the relationship between physicals signs and mutation subgroups with CVEs, reporting hazard ratios (HRs), 95% CIs and *p* for trend. Two models were employed to test these relationships. The crude model was unadjusted. The adjusted model was controlled for age, sex, BMI, CAD, smoking, hypertension, DM, family history of CAD, adjusted LDL-C levels, lipid-lowering therapy, LDL cholesterol year score and total cholesterol year score. *p* values < 0.05 were considered statistically significant. Analyses were performed using IBM SPSS Statistics version 25.0 (IBM SPSS Statistics, IBM Corp., Armonk, NY, USA) and R (http://www.r-project.org/) statistical packages.

## Results

### Clinical characteristics

Of the 466 subjects, 55 (11.8%) exhibited physical signs. PS matching yielded a cohort of 250 subjects, and 50 patients with TX/CA were matched at a 4:1 ratio to patients without TX/CA. Baseline characteristics of patients are shown in Table 1. The mean age of the study cohort was 47.8 ± 12.6 years, and 62% were males. Patients with TX/CA had similar prevalence of BMI, prevalence of CAD, number of diseased vessels and traditional cardiovascular risk factors compared with those without.

**Table 1:** Baseline characteristics of the study population

	Propensity matched 1:4			
	Total (n = 250)	TX/CA (n = 50)	Non-TX/CA (n = 200)	*p* value
*Demographic parameters*				
Age, year	47.8 ± 12.6	47.9 ± 13.0	47.7 ± 12.5	0.895
Men, % (n)	62.2 (140)	66.7 (30)	61.1 (110)	0.492
*Coronary risk factors*				
BMI, kg/m^2^	25.1 ± 3.31	24.9 ± 3.43	25.1 ± 3.29	0.731
HT, % (n)	34.2 (77)	28.9 (13)	35.6 (64)	0.399
DM, % (n)	16.4 (37)	11.1 (5)	17.8 (32)	0.281
HC, % (n)	80.9 (182)	88.9 (40)	78.9 (142)	0.127
Smoking, % (n)	37.8 (85)	35.6 (16)	38.3 (69)	0.731
Family history of CAD, % (n)	37.3 (84)	35.6 (16)	37.8 (68)	0.783
*Lipid parameters*				
TC, mmol/L	7.19 ± 2.24	7.66 ± 2.64	7.06 ± 2.11	0.167
TG, mmol/L	1.51 (1.15–2.13)	1.26 (1.09–1.56)	1.60 (1.22–2.30)	**0.001**
LDL-C, mmol/L	5.34 ± 1.95	5.94 ± 2.38	5.19 ± 1.80	0.051
Uncontrolled LDL-C, mmol/L	7.90 ± 2.28	8.65 ± 2.53	7.70 ± 2.18	**0.025**
HDL-C, mmol/L	1.11 ± 0.37	1.00 ± 0.34	1.14 ± 0.37	**0.021**
NHDL-C, mmol/L	6.04 ± 2.17	6.66 ± 2.69	5.87 ± 1.99	0.072
Apo A1, g/L	1.26 ± 0.33	1.11 ± 0.37	1.30 ± 0.31	**0.001**
Apo B, g/L	1.46 ± 0.44	1.60 ± 0.57	1.42 ± 0.39	0.064
Lp (a), mg/L	325.1 (169.2–710.4)	394.0 (158.8–668.3)	301.1 (173.6–739.0)	0.792
FFA, mg/dL	0.49 ± 0.46	0.50 ± 0.20	0.49 ± 0.50	0.851
Total cholesterol year score	12,794 ± 5878	13,394 ± 5379	12,646 ± 5997	0.787
LDL cholesterol year score	9549 ± 4605	10,379 ± 4511	9345 ± 4616	0.498
*Genetic spectrum*				
Definite FH (DLCN score > 8), % (n)	57.6(144)	94.0(47)	48.5(97)	**< 0.001**
Probable FH (DLCN score 6–8), % (n)	42.4(106)	6.0(3)	51.5(103)	**< 0.001**
Mutation positive, % (n)	51.2 (128)	72.0 (36)	38.0 (76)	**0.001**
*LDLR* (+) mutation, % (n)	38.8 (97)	66.0 (33)	32.0 (64)	**< 0.001**
1 allele	26.8 (67)	42.0 (21)	23.0 (46)	**0.007**
2 or more alleles	12.0 (30)	24.0 (12)	9.0 (18)	**0.004**
*LDLR* (−) mutation, % (n)	10.8 (27)	6.0 (3)	12.0 (24)	0.221
*APOB* (+), % (n)	7.2 (18)	4.0 (2)	8.0 (16)	0.328
*PCSK9* (+), % (n)	3.6 (9)	2.0 (1)	4.0 (8)	0.497
Double or triple mutation ( +), % (n)	1.6 (4)	0 (0)	2.0 (4)	0.313
*Incidence of coronary atherosclerosis*				
CAD, % (n)	72.0 (180)	78.0 (39)	71.1 (128)	0.132
Diseased vessels, % (n)	62.8 (157)	62.2 (28)	60.6 (109)	0.838
SVD, % (n)	12.4 (31)	6.7 (3)	12.2 (22)	0.289
DVD, % (n)	17.6 (44)	15.6 (7)	20.0 (36)	0.498
MVD, % (n)	32.8 (82)	40.0 (18)	28.3 (51)	0.129
*Coronary severity*				
Gensini score	28.0 (8.0–56.0)	48.5 (20.0–79.5)	26.5 (6.0–49.8)	**0.011**
SYNTAX score	9.0 (3.8–17.3)	16.0 (9.8–25.1)	9.0 (2.0–15.0)	**< 0.001**
Jeopardy score	4.0 (1.5–6.0)	5.0 (2.0–6.0)	2.0 (0–5.5)	**0.032**
*In hospital drug treatment*				
Aspirin, % (n)	71.1 (160)	66.7 (30)	72.2 (130)	0.462
P2Y12 inhibitor, % (n)	21.3 (48)	42.2 (19)	16.1 (29)	**< 0.001**
ACEI/ARB, % (n)	28.0 (63)	28.9 (13)	27.8 (50)	0.882
β-blockers, % (n)	56.0 (126)	53.3 (24)	56.7 (102)	0.687
CCB, % (n)	21.8 (49)	20.0 (9)	22.2 (40)	0.747
Statins, % (n)	82.2 (185)	84.4 (38)	81.7 (147)	0.663
Ezetimibe, % (n)	48.4 (109)	62.2 (28)	45.0 (81)	**0.039**

Data shown are mean ± SD, median (Q1–Q3 quartiles) or n (%). Bold values indicate statistical significance

FH: familial hypercholesterolemia; TX: tendon xanthomas; CA: corneal arcus; BMI: body mass index; HT: hypertension; DM: diabetes mellitus; HC: hypercholesterolemia; CAD: coronary artery disease; TC: total cholesterol; TG: triglyceride; LDL-C: low density lipoprotein cholesterol; HDL-C: high density lipoprotein cholesterol; NHDL-C: non-high density lipoprotein cholesterol; Apo A1: apolipoprotein A1; Apo B: apolipoprotein B; Lp(a): lipoprotein(a); FFA: free fatty acid; DLCN: Dutch Lipid Clinic Network; *LDLR*: low-density lipoprotein receptor; *APOB*: apolipoprotein B; *PCSK9*: proprotein convertase subtilisin/kexin type 9; *SVD*: single vessel disease; *DVD*: double vessels disease; *MVD*: multiple vessels disease; *ACEI*: angiotensin converting enzyme inhibitor; *ARB*: angiotensin receptor blockers; *CCB*: calcium channel blockers

During hospitalization, statin treatment did not differ between the two groups, and ezetimibe and P2Y12 inhibitors were prescribed more in the TX/CA group. No differences in the prescription of aspirin, ACEI/ARB, β-blockers and CCB were found between patients with and without physical signs.

### Genetic spectrum

In our study cohort, the proportion of the definite (DLCN > 8) and probable (DLCN 6–8) FH was 57.6% and 42.4%, respectively. Positive mutations in *LDLR*, *APOB* and *PCSK9* were found in 128 patients (51.2%), with 36 patients (72.0%) in the TX/CA group and 76 patients (38.0%) in the non-TX/CA group. *LDLR* mutation carriers were 97 (38.8%). As expected, the prevalence of *LDLR* mutations was significantly higher in the TX/CA group than in the non-TX/CA group (66.0% vs. 32.0%, *p* < 0.001). In addition, the proportion of numbers of alleles also showed statistical significance between the two groups. However, the prevalence of *APOB*, *PCSK9* and double or triple mutations showed no difference between patients with and without physical signs (Table 1). Spectrum and frequency of pathogenic mutations in patients with or without TX/CA were depicted in Additional file 1: Fig. S2.

We further performed a regression analysis to determine the association between genetic mutations and physical signs. Compared with subjects without mutations, subjects with *LDLR* positive mutations had a threefold higher odds ratio of TX/CA (OR 3.98; 95% CI 1.98–7.99; *p* < 0.001). After adjusting for cardiovascular risk factors, including uncontrolled LDL-C levels, total cholesterol year score and LDL cholesterol year score, the trend did not change (OR 3.49; 95% CI 1.44–8.47; *p* = 0.006; Table 2, Additional file 1: Fig. S2).

**Table 2:** Regression analyses to assess the association between TX/CA and genetic mutations in patients with HeFH after propensity score matching

Characteristics	No mutation	*LDLR* (−)	*LDLR* (+)
*Model 1 ORs*	1 (Reference)	0.827 (0.222–3.077)	3.978 (1.980–7.989)
*p* value		0.776	** < 0.001**
*Model 2 ORs*	1 (Reference)	0.810 (0.217–3.021)	4.193 (2.060–8.534)
*p* value		0.754	** < 0.001**
*Model 3 ORs*	1 (Reference)	0.919 (0.190–4.451)	3.493 (1.442–8.465)
*p* value		0.917	**0.006**

Model 1 unadjusted, model 2 age- and sex- adjusted, model 3 fully adjusted for age, sex, BMI, CAD, smoking, hypertension, DM, family history of CAD, uncontrolled LDL-C levels, lipid-lowering therapy, total cholesterol year score and LDL cholesterol year score. Bold values indicate statistical significance

*LDLR*: low-density lipoprotein receptor; *LDLR* (−): LDLR negative mutation; *LDLR* (+): LDLR positive mutation; *OR*: odds ratio; *BMI*: body mass index; *CAD*: coronary artery disease; *DM*: diabetes mellitus

### Lipid profile, lipid- and inflammation-related markers

As depicted in Table 1, the LDL-C levels before lipid-lowering treatment were significantly higher in the TX/CA group than in the non-TX/CA group (8.65 ± 2.53 vs. 7.70 ± 2.18 mmol/L, *p* = 0.025). There were no differences in the levels of TC, non-HDL-C and ApoB between the two groups. However, the levels of TG, HDL-C and ApoA1 were lower in patients with TX/CA than those without TX/CA (*p* < 0.01).

The results for lipid- and inflammation-related markers also showed a significant difference between tertiles, and the prevalence of TX/CA was significantly higher in patients with higher tertiles of PCSK9 and hsCRP levels (*p* = 0.004 and *p* = 0.046, respectively). Of note, the prevalence of TX/CA in patients was not significantly different when considering tertiles of Lp(a) levels (*p* = 0.807, Additional file 1: Figure S3).

### CAD severity and coronary calcifications

**Fig. 2:**
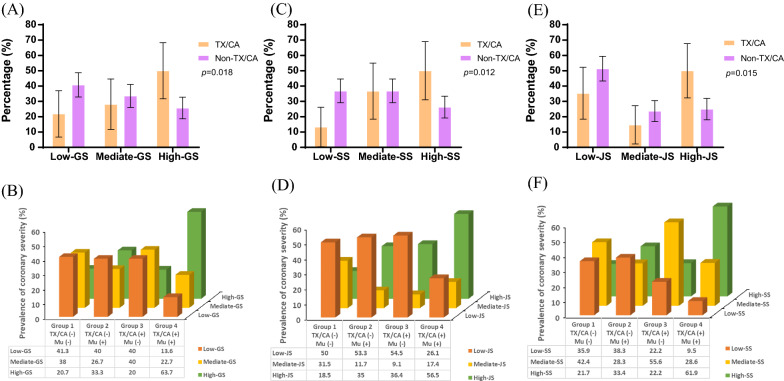
Prevalence of coronary severity in HeFH patients. The bars represent the 95% confidential interval for each percentage. Different tertiles of Gensini (**A**), SYNTAX (**B**), and Jeopardy (**C**) scores were compared between patients with and without TX/CA. Different tertiles of Gensini (**D**), SYNTAX (**E**), and Jeopardy (**F**) scores were also compared among patients in four mutation subgroups (group1: TX/CA(−), Mu(−); group2: TX/CA(−), Mu(+); group3: TX/CA(+), Mu(−); group4: TX/CA(+), Mu(+)). TX, tendon xanthomas; CA, corneal arcus; GS, Gensini score; SS, SYNTAX score; JS, Jeopardy score; Mu: mutation; Mu(+): mutation positive; Mu(−): mutation negative

Physical signs were associated with the severity of coronary disease. The median (Q1–Q3 quartiles) values for the Gensini, SYNTAX and Jeopardy scores were 28.0 (8.0–56.0), 9.0 (3.8–17.3) and 4.0 (1.5–6.0), respectively. As the tertiles of the three scores increased, the TX/CA group demonstrated significantly higher tertiles of Gensini, SYNTAX and Jeopardy scores than the non-TX/CA group (*p* = 0.018, *p* = 0.012 and *p* = 0.015, respectively; Fig. 2A, C and E). Analysis of genetic subgroups revealed that there were significantly more patients in group 4 (physical signs and genetic mutations both positive) in higher tertiles of Gensini, SYNTAX, and Jeopardy scores than in groups 1, 2, and 3 (physical signs and genetic mutations both negative, with physical signs, or one of the two being positive; *p* = 0.007, *p* = 0.008, and *p* = 0.002, respectively; Fig. 2B, D and F).

CAC score analysis was conducted in a total of 77 subjects to substantiate the finding of the Mann–Whitney U analysis, which indicated that CAC scores were significantly higher in patients with physical signs than in those without them (27 [0–190] vs. 0 [0–26], *p* = 0.041).

### Survival analysis and CVE outcomes

**Fig. 3:**
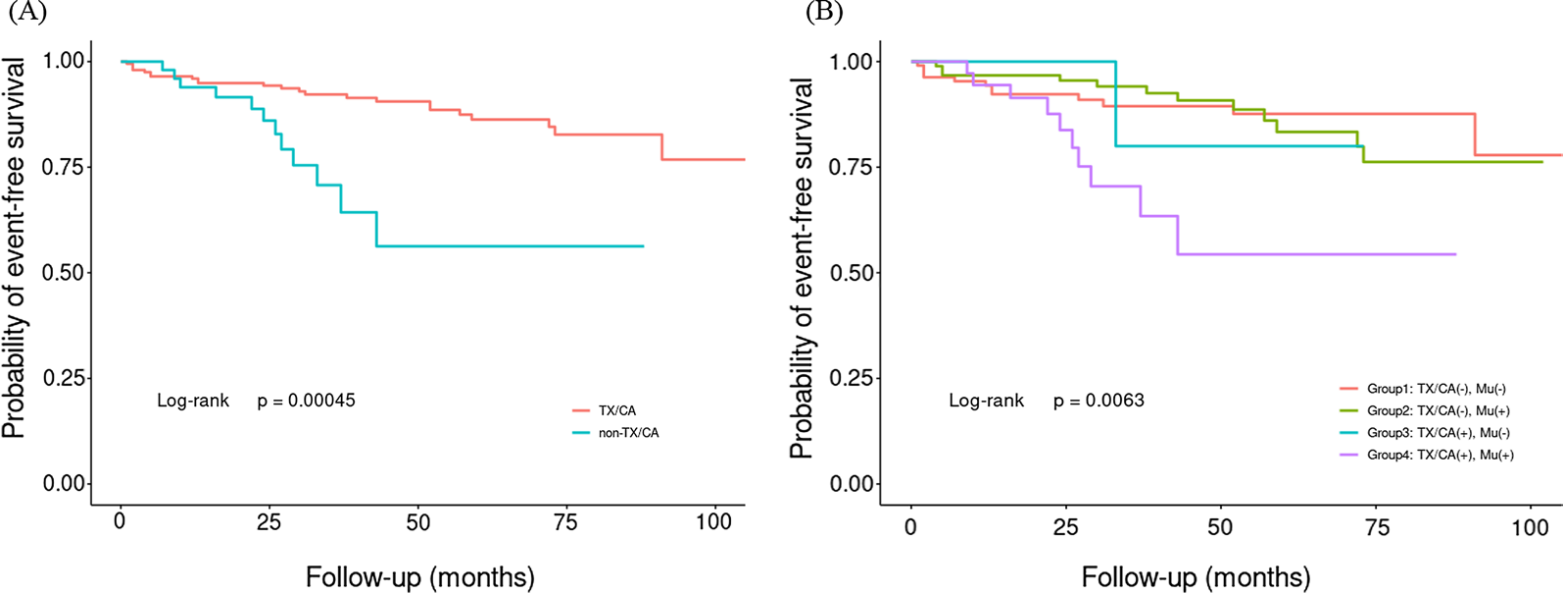
The Kaplan–Meier plot of cardiovascular outcomes in patients with HeFH. **A** The cumulative event-free survival analysis in patients with and without physical signs (TX/CA); **B** the cumulative event-free survival analysis among four mutation subgroups (group1: TX/CA(−), Mu(−); group2: TX/CA(−), Mu(+); group3: TX/CA(+), Mu(−); group4: TX/CA(+), Mu(+)). TX: tendon xanthomas; CA: corneal arcus; Mu: mutation; Mu(+): mutation positive; Mu(−): mutation negative

Over an average of 3.7 years of follow-up, 35 CVEs were recorded, including 6 cardiovascular deaths, 4 non-fatal MI, 9 strokes, and 16 coronary revascularizations. Kaplan–Meier curves showed that the TX/CA group had a significantly lower cumulative event-free survival rate compared with the non-TX/CA group (*p* < 0.001; Fig. 3A). The survival rates also showed a significant difference among the four genetic subgroups (*p* < 0.01; Fig. 3B).

We further assessed the predictive performance of the physical signs, and patients in the TX/CA group had a 2.8-fold higher risk of CVEs in the crude model than those in the non-TX/CA group. After adjusting for traditional risk factors including family history of CAD, uncontrolled LDL-C levels, total cholesterol year score and LDL cholesterol year score, the risk was still two to three times higher in the TX/CA group than in the non-TX/CA group and statistically significant (HR, 2.75; 95% CI 1.04‒7.26, *p* = 0.024). Among the four genetic subgroups, the risk in group 4 was 3 times higher than in group 1 (HR 3.34; 95% CI 1.04–10.72, *p* = 0.024; Table 3).

**Table 3:** Cox Regression Models in predicting cardiovascular outcomes according to physical signs

CVEs	Events, n/total	Hazard ratio (95% CI)	
		Crude model	Adjusted model
Non-TX/CA	24/200	1 (reference)	1 (reference)
TX/CA	11/50	2.86 (1.37–5.98)**	2.75 (1.04–7.26)*
Group1 TX/CA (−) Mu (−)	12/108	1 (reference)	1 (reference)
Group2 TX/CA (−) Mu (+)	12/92	1.07 (0.48–2.39)	0.82 (0.34–1.98)
Group3 TX/CA (+) Mu (−)	1/14	1.01 (0.13–7.89)	1.02 (0.12–8.49)
Group4 TX/CA (+) Mu (+)	10/36	3.66 (1.55–8.64)**	3.34 (1.04–10.72)*

Adjusted model was adjusted for age, sex, BMI, CAD, smoking, hypertension, DM, family history of CAD, adjusted LDL-C levels, lipid-lowering therapy, LDL cholesterol year score and total cholesterol year score

TX: tendon xanthomas; CA: corneal arcus; Mu: mutation; Mu (+), mutation positive; Mu (−), mutation negative

*p < 0.05, **p < 0.01

## Discussion

Intriguingly, in the present study we found that the presence of physical signs was significantly associated with a higher LDL-C level, higher PCSK9 or hsCRP level, and more *LDLR* positive mutations. Additionally, patients in the TX/CA group suffered from more severe coronary lesions and calcification than those in the non-TX/CA group. The proportion of patients with TX/CA was higher for higher tertiles of Gensini, SYNTAX, and Jeopardy scores as well as CAC scores. More importantly, subjects presenting with physical signs had a higher mutation-positive rate accompanied by a higher risk of CVEs. Thus, our data may provide novel information regarding the potential effects of TX/CA on genotype, coronary disease severity, coronary calcification, and cardiovascular outcomes in a Chinese HeFH cohort.

Physical signs (TX and/or CA) are highly frequent in homozygous forms and occur in less than 15% of contemporary heterozygous FH populations [24]. Pathological studies define xanthomas as cholesterol depots, foam cell formation and accumulation of connective tissue on dermal and tendon tissues. One previous study has associated the presence of xanthomas with genes related to reverse cholesterol transport and LDL oxidation pathway [12]. Previous evidence had associated presence of xanthomas with higher LDL-C, male sex and hypertension, and especially with greater frequency of previous CVD in molecularly confirmed FH individuals [25]. Tada et al. also reported that the clinical signs of FH (xanthomas and/or family history of CAD) and a positive FH mutation status additively increased the risk for CAD in patients with FH [11]. Wong et al. suggested that CA was associated with incident cardiovascular disease, independent of serum lipids and traditional risk factors, though the subjects included were not FH patients [10]. In addition, it was observed that the appearance of xanthomas was related to the presence of traditional risk factors and to the concentration of LDL-C in 394 Japanese patients with CAD [26]. Hence, the presence of xanthomas and possibly corneal arcus are considered markers of exposure to higher LDL-C and indicators greater CVD risk in patients with HeFH. In our study, we evaluated the conventional risk factors and found that uncontrolled (adjusted) LDL-C levels were associated with TX/CA. Of note, patients presenting with TX/CA had a 2- to threefold higher risk of CVEs, indicating that physical signs are clinically relevant and may help in risk stratification in patients with HeFH.

Of additional importance, patients with physical signs had higher PSCK9 levels than Lp(a) levels, which may explain the rapid regression of physical signs by PCSK9 inhibitors. Previous studies have demonstrated that treatment with PCSK9 inhibitors lowered plasma LDL-C levels by about 50% and led to an increase in hepatic LDL receptor function, resulting in the reversal of lipid accumulation in patients with FH [27]. Moreover, Bea et al. reported that prolonged PCSK9 inhibition in HeFH promoted xanthomas regression more than high doses of potent statins, consistent with evidence regarding the beneficial effects of PCSK9 inhibitors in the treatment of atherosclerosis [28]. Hence, the introduction of PCSK9 inhibitors provides new therapeutic options and promotes the management of patients with HeFH with physical signs. In addition, inflammation-related markers, such as hsCRP, have also been demonstrated as key risk factors for atherosclerosis, and CRP is deposited in the arterial wall during atherogenesis [7]. Our study found that higher hsCRP levels occur in parallel with the appearance of physical signs, which may reveal a novel aspect of the pathogenesis of atherosclerosis.

Notably, physical signs are also strongly associated with a genetic diagnosis and are highly specific for FH patients. According to the Simon Broome Register criteria and the MedPed criteria [29], the presence of physical signs is an important distinguishing feature of FH. In addition, it was reported that physical signs are clustered in families; therefore, it is reasonable to speculate that mutations might be responsible for hypercholesterolemia and play a role in the development of FH. Oosterveer et al. showed that the presence of xanthomas in patients with FH is associated with genetic variation in reverse cholesterol transport and LDL oxidation pathways [30]. However, the association between the presence of physical signs and FH genetic spectrum has not been fully evaluated. Genetically, in our study population, we found that the positive mutation rate is significantly higher in the TX/CA group than the non-TX/CA group (72% vs. 38%). Moreover, patients with genetic mutations along with physical signs had a threefold higher CVE risk than those without genetic mutations or signs. Based on these facts, it may be more necessary for patients with TX/CA to access genetic testing. The utility of FH genetic testing could facilitate diagnosis, identify higher cardiovascular risk which indicates the potential need for more aggressive lipid lowering therapy, and could probably promote cascade testing of at-risk relatives. Thus, our study provided evidence associated with intensions to have FH genetic testing among patients with physical signs.

Though a pilot study has reported the association between TX/CA and the extents of subclinical atherosclerosis and coronary calcification quantified by both tomographic scores and coronary angiography in FH patients [31], few studies have assessed the relationship between xanthomas and angiographic severity and the extent of CAD in FH patients. Kitahara et al. assessed CAD severity using the SYNTAX score, and found that the SYNTAX score progressively increased with an increase in greater Achilles tendon thickness [26]. Besides the SYNTAX score, we evaluated the Gensini and Jeopardy scores to identify angiographic severity in patients with HeFH. In our analysis, patients with physical signs showed a higher prevalence of high tertiles of Gensini, SYNTAX, and Jeopardy score; hence, detecting physical signs may be useful for the identification of patients with advanced CAD.

There are several limitations to this study that merit attention. First, our study was a single-center study, and the sample size was relatively small, especially in patients with physical signs. This may have introduced bias in the analysis. This is why we used propensity matching, to minimize the confounding because of the differences in age and sex. Second, we could not determine the concentration of LDL-C during the follow-up period. It would be more accurate to determine lipid levels using a semi-annual follow-up period. Besides, we used the adjusted LDL-C level as a replacement to improve the accuracy since the LDL-C level before admission was unknown. Third, there are several compound heterozygotes in our cohort, and we did not to further elaborated classification due to the small sample size. Finally, higher cholesterol burden usually accompanies presence of physical signs in FH, hence we calculated the cholesterol year score and adjusted results for it.

In conclusion, in patients with HeFH, physical signs (TX/CA) were positively correlated with positive genetic mutation, higher PCSK9 or hsCRP concentration, severity of coronary stenosis or calcification and independently associated with worse outcomes in case of clinically diagnosed FH, suggesting that physical signs may help in risk stratification of and developing treatment strategies for patients with HeFH.

## Supplementary Information

**Additional file 1.** Additional figures and Tables.

## Data Availability

The datasets used and/or analyzed during the current study are available from the corresponding author on reasonable request.

## Abbreviations

FH: Familial hypercholesterolemia
HeFH: Heterozygous familial hypercholesterolemia
LDL-C: Low-density lipoprotein cholesterol
TX: Tendon xanthomas
CA: Corneal arcus
CAD: Coronary artery disease
*LDLR*: LDL receptor
*APOB*: Apolipoprotein B
*PCSK9*: Proprotein convertase subtilisin/kexin type 9
Lp(a): Lipoprotein (a)
hsCRP: High-sensitivity C-reactive protein
PS: Propensity score
CVEs: Cardiovascular events
DLCN: Dutch Lipid Clinic Network
TC: Total cholesterol
TG: Triglyceride
HDL-C: High-density lipoprotein cholesterol
MI: Myocardial infarction
BMI: Body mass index
T2DM: Type 2 diabetes mellitus
CAC: Coronary artery calcium
GS: Gensini score
SS: SYNTAX score
JS: Jeopardy score
