# Supplementary material for: Relations of physical signs to genotype, lipid and inflammatory markers, coronary stenosis or calcification, and outcomes in patients with heterozygous familial hypercholesterolemia
Source: J Transl Med. 2021 Dec 7;19:498. doi: 10.1186/s12967-021-03166-w (PMC8650321; doi:10.1186/s12967-021-03166-w)
Supplement: Supplementary file 1 — Additional file 1. Additional figures and Tables. [file 12967_2021_3166_MOESM1_ESM.docx]

**Supplementary Table S1**. Covariate balance between TX/CA and non-TX/CA groups before and after propensity score matching.

| Variable | Absolute standardized mean differences | |
| --- | --- | --- |
|  | Before matching | After matching |
| All cases (propensity) | 0.299 | 0.015 |
| Sex | 0.117 | 0.029 |
| Age | -0.280 | -0.008 |

**Supplementary Table S2**. Cholesterol year scores of the study population.

| Variable | Total  (n=250) | TX/CA  (n=50) | Non-TX/CA  (n=200) | p value |
| --- | --- | --- | --- | --- |
| LDL cholesterol year score  ≤6000, % (n)  6000-16000, % (n)  >16000, % (n) | 9549 ± 4605  19.6% (49)  69.6% (174)  10.8% (27) | 10379 ± 4511  16.0% (8)  68.0% (34)  16.0% (8) | 9345 ± 4616  20.5% (41)  70.0% (140)  9.5% (19) | 0.498  0.331  0.891  0.292 |
| Total cholesterol year score  ≤6000, % (n)  6000-16000, % (n)  >16000, % (n) | 12794 ± 5878  5.2% (13)  71.6% (179)  23.2% (58) | 13394 ± 5379  4% (2)  66% (33)  30% (15) | 12646 ± 5997  5.5% (11)  73.0% (146)  21.5% (43) | 0.787  0.355  0.404  0.288 |

LDL cholesterol year score was calculated by the highest total cholesterol value by the patient’s age at clinical diagnosis, to which was added the LDL-C value at the inclusion visit in the registry multiplied by the time spent until the inclusion visit, as follows: LDL-C max (mg/dL) at diagnosis * age at diagnosis + LDL-C (mg/dL) at inclusion * [age at inclusion – age at diagnosis]. The total cholesterol year score was calculated as: total cholesterol year score (mg-year/dL) = TC max (mg/dL) at diagnosis * age at diagnosis + TC (mg/dL) at inclusion * [age at inclusion – age at diagnosis].

**Supplementary Figure S1.** Flowchart of the study population. CAD, coronary artery disease; FH, familial hypercholesterolemia; DLCN, Dutch Lipid Clinic Network ;HeFH, heterogeneous familial hypercholesterolemia; HoFH, homogeneous familial hypercholesterolemia; TX, tendon xanthomas; CA, corneal arcus; Mu, mutation; Mu(+), mutation positive; Mu (-), mutation negative.

**Supplementary Figure S2.** Spectrum and frequency of pathogenic mutations in HeFH cohort between TX/CA group (A) and non-TX/CA group (B). All mutations were named with gene and cDNA. Others indicates that numbers of carriers are less than two individuals.

**
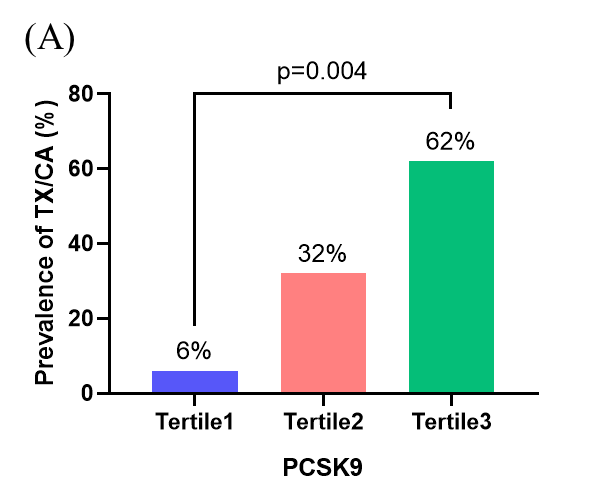
**

**
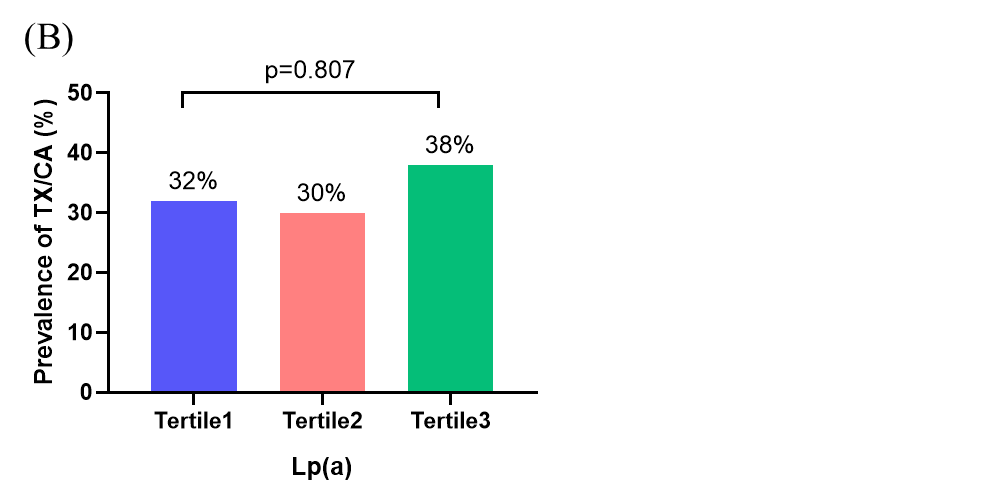
**

**
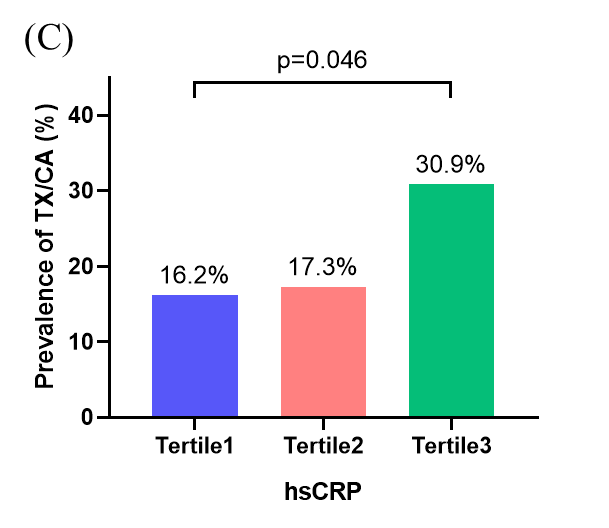
**

**Supplementary Figure S3.** Association between lipid- and inflammation-related markers with TX/CA. Markers include PCSK9 (A), Lp(a) (B), and hsCRP (C). TX, tendon xanthomas; CA, corneal arcus; PCSK9, proprotein convertase subtilisin/kexin type 9; Lp(a), lipoprotein (a); hsCRP, high-sensitivity C-reactive protein.
